# Cardiac Pain and Angina Pectoris

**Published:** 1897-12-04

**Authors:** S. H. Habershon

**Affiliations:** Assistant Physician and Pathologist to the Hospital


### Medical Progress and Hospital Clinics.

[*The Editor will be glad to receive offers of co-operation and contributions from members of the profession. All letters should be addressed to* The Editor, at the Office, 28 & 29, Southampton Street, Strand, London, W.C.]

### CARDIAC PAIN AND ANGINA PECTORIS.

A Clinical Lecture and Demonstration given at the Brompton Hospital for Consumption and Diseases of the Chest, by S. H. Habershon, M.D., F.R.C.P., Assistant Physician and Pathologist to the Hospital.

(*Continued from page* 151.)

**Cardiac Valvular Disease.—Aortic Stenosis or Dilatation.**—Cardiac pain in connection with valvular disease or aneurism near the valves is extremely common, and we are all familiar with the cases. I have several here to-day. In the more severe forms the symptoms differ very little from a chronic angina pectoris occurring in a heart without manifest cardiac lesion.

The patient, T. C., is a man aged 51, a teacher of music. He had rheumatic fever 24 years ago, and again very severely six years ago. For two or three years he has suffered from severe paroxysmal pain in the chest, worse at night, and often waking him from sleep. The same incidents bring on the pain in the daytime as in the previous case. This patient illustrates one point of great importance in its bearing on the diagnosis. He applied to his doctor first for pain after food, and for a long time was treated for dyspepsia. Eventually it was recognised that the pain was cardiac, and he was sent to see Dr. Sansom.

It is exceedingly common to find in the histories of patients who suffer from cardiac pain that they first describe the pain as produced by food, but a careful inquiry will elicit the fact that, if they sit still after food, there is no pain, but it is exertion on a full stomach that excites the cardiac spasm.

You will find that the patient has a large hypertrophied heart, a greatly increased area of dulness in all directions, impairment of resonance in the second right intercostal space near the sternum, and a loud double bruit over the aorta, heard especially loudly about one inch beyond the right sternal margin. There is no pulsation to be felt, and I have not suspected aneurism. He has undoubted aortic valvular disease with dilatation of the first part of the aorta. The pulse is a marked example of the water-hammer pulse. He visited me on October 16th, and while sitting in his chair an attack of cardiac pain came on. The heart had previously been beating quietly. The pulse now became violent and of considerable tension—132 beats per minute during the height of the attack, gradually becoming 108, and finally 88 as the attack passed off, after eating a nitroglycerine lozenge. The breathing was shallow and sighing, the face anxious and with a pinched expression, the forehead clammy. The whole paroxysm was over in less than ten minutes. This patient has numerous attacks, and we estimate the severe ones by the number of tabloids of nitroglycerine he is obliged to take. At first he was taking twenty-six to twenty in the day and night, now he is taking rather fewer. The pain is referred to the lower part of the sternum, but radiates to the left arm.

The patient presents to you a very severe form of angina pectoris associated with valvular disease.

There are some five or six other patients here with cardiac pain of varying degree of severity, all with aortic disease or aortic dilatation.

The pathological evidence at our disposal is ample, and it is usually found that the endocardial inflammation and thickening has extended above the aortic valves, or that there is atheroma of the aorta involving the coronary orifices. In cases of dilated aorta not dependent on valvular disease, but due to atheromatous degeneration, we see sometimes the openings extremely constricted. In the specimen I show you, taken from the body of a man who died a few days ago, there is to be found extensive atheroma of all the cardiac vessels, and calcareous patches are felt in the main coronary branches. The hypertrophied cardiac muscle is the seat of patchy fibroid change. The aortic valves, as well as the mitral, are thickened, and the former are puckered and incompetent. The man suffered from cardiac pain of moderate intensity, for which he was given nitroglycerine tabloids.

**Aneurism and Spurious Angina.**—Sometimes in cases of dilated aorta or aneurism there appears to be direct pressure upon the cardiac plexuses at the base of the heart. Douglas Powell relates two remarkable cases, one of direct, another of reflex irritation. The former, a case of tuberculous pericarditis in a woman, who was the subject of chronic phthisis. The other, a man who suffered from renal calculus. In both the paroxysms of pain eventually passed off. Lastly, aneurismal and other tumours will produce severe spasms of cardiac pain. One case I well remember in St. Bartholomew's Hospital; the cause was a small sacculated aneurism, which eventually burst into the left bronchus. To these cases the term *spurious* or *pseudo-angina* may fairly be applied.

**Distension of Cardiac Cavities in Mitral Disease.**—In one other class of cases, which is not generally recognised, there is a severe cardiac pain in all its characters resembling angina pectoris

It has usually been said that patients with mitral disease do not suffer from this form of cardiac pain. I have seen two cases, and one patient is present to-day.

Ada N., whose case I referred to early in my lecture, is the subject of mitral disease. She has a large hypertrophied heart, and for five or six years has suffered from a continuous cardiac pain, greatly increased by exertion. For the last few months, however, a severe spasmodic pain has appeared, coming on after exertion, shooting down the left arm to the fingers, producing anxiety and dread, and not differing from the other forms of angina that have been described.

It has been so frequently stated by various authors that angina pectoris is not seen in association with mitral disease, and that when the mitral valves become incompetent in a typical case the pain is relieved, that I feel in some doubt as to the true pathology of this pain. A case I saw some years ago seems to me to throw some light upon it. A young girl, aged about 15, who suffered from mitral disease, was seized with sudden severe cardiac pain affecting the whole præcordial area. The pain was very intense, and was relieved by nitrite of amyl. I am not able to obtain the notes of the case, and cannot at this moment give a careful record. The important point appeared to be that the pain was not gastric, and was accompanied by a sudden and extreme dilatation of heart, which gradually diminished after the attack was relieved. The inducing cause of the paroxysm was mental shock. The pain, as far as my recollection goes, did not extend to the left arm. This would seem to indicate, and the opinion was expressed at the time, that distension of the cardiac cavities is capable of producing severe pain, in some respects similar to angina pectoris. Whether this is so in the case of A. N., who is shown to you, I cannot say.

I shall now proceed to show you the few specimens I have obtained from our museum, and to demonstrate the cases here for your inspection. I need scarcely beg you to be careful not to fatigue the patients, and to percuss the chest very lightly.

